# Neck Muscle EMG-Force Relationship and Its Reliability During Isometric Contractions

**DOI:** 10.1186/s40798-017-0083-2

**Published:** 2017-04-14

**Authors:** Riccardo Lo Martire, Kristofer Gladh, Anton Westman, Björn O. Äng

**Affiliations:** 1 Division of Physiotherapy, Department of Neurobiology, Care Sciences, and Society, Karolinska Institutet, Alfred Nobels allé 23 100, Huddinge, 141 83 Sweden; 2 Department of Physiology and Pharmacology, Karolinska Institutet, Stockholm, Sweden; 3 Department of Aeronautical and Vehicle Engineering, KTH Royal Institute of Technology, Stockholm, Sweden; 4 Department of Anesthesia and Intensive Care, Karolinska University Hospital, Huddinge, Sweden; 5 School of Education, Health and Social Studies, Dalarna University, Falun, Sweden

**Keywords:** Biomechanics, Cervical spine, Electromyography, Moment, Muscle activity, Semispinalis capitis, Splenius capitis, Sternocleidomastoideus, Torque

## Abstract

**Background:**

Susceptible to injury, the neck is subject to scientific investigations, frequently aiming to elucidate possible injury mechanisms via surface electromyography (EMG) by indirectly estimating cervical loads. Accurate estimation requires that the EMG-force relationship is known and that its measurement error is quantified. Hence, this study examined the relationship between EMG and isometric force amplitude of the anterior neck (AN), the upper posterior neck (UPN), and the lower posterior neck (LPN) and then assessed the relationships’ test-retest reliability across force-percentiles within and between days.

**Methods:**

EMG and force data were sampled from 18 participants conducting randomly ordered muscle contractions at 5–90% of maximal voluntary force during three trials over 2 days. EMG-force relationships were modeled with general linear mixed-effects regression. Overall fitted lines’ between-trial discrepancies were evaluated. Finally, the reliability of participants’ fitted regression lines was quantified by an intraclass correlation coefficient (ICC) and the standard error of measurement (SEM).

**Results:**

A rectilinear model had the best fit for AN while positively oriented quadratic models had the best fit for UPN and LPN, with mean adjusted conditional coefficients of determination and root mean square errors of 0.97–0.98 and 4–5%, respectively. Overall EMG-force relationships displayed a maximum 6% between-trial discrepancy and over 20% of maximal force, and mean ICC was above 0.79 within day and 0.27–0.61 between days across areas. Corresponding SEM was below 12% both within and between days across areas, excluding UPN between days, for which SEM was higher.

**Conclusions:**

EMG-force relationships were elucidated for three neck areas, and provided models allow inferences to be drawn from EMG to force on a group level. Reliability of EMG-force relationship models was higher within than between days, but typically acceptable for all but the lowest contraction intensities, and enables adjustment for measurement imprecision in future studies.

## Key Points

- Of the studied relationships, a linear relationship was best suited for the anterior neck while exponential relationships were best suited for the upper and lower posterior neck.
- Overall EMG-force relationships were similar between trials which suggests that they are stable over time.
- Provided models can be used to draw inferences from EMG to force on a group level.

## Background

The human neck allows head movements and load-bearing while protecting vital neural structures and stabilizing the visual and vestibular systems [1]. Its flexibility [2], combined with the heavy head weight relative to the neck muscles’ force capacity [3, 4], however, makes the area susceptible to pain and injury. Neck pain is the fourth leading cause of disability globally [5], with an average one-year prevalence of 37% in the general population [6]. During athletics, where rapidly changing conditions result in high demands on the spine, the neck is also prevalently injured, sometimes gravely [7–11]. Consequently, the area is subject to scientific investigations, not least within the sport sciences [12–14].

From a functional perspective, the neck can be viewed as a three-part stabilizing system in which support is provided by passive spinal structures, while stability is regulated according to demands via the neck muscles in a feedback-driven neural control scheme [1]. To coordinate swift variations in spinal posture and loads, a highly optimized system which stabilizes the spine in both static and dynamic situations is required [1]. Two neural control strategies have been identified for maintaining cervical spine stability in an upright neutral posture: reciprocal muscle activation, which is direction-specific to postural perturbations, and co-contraction of agonistic and antagonistic muscles [15]. In the sagittal plane, these strategies presumably function primarily via four main neck force-generating muscles, which include the sternocleidomastoids, the semispinalis capitis, and the splenius capitis [2]. These muscles are all near their maximum force-generating capacity in a neutral spine posture [2], wherein perturbations are commonly countered under quasi-isometric conditions [15], and are frequently targeted in studies of the neck [12–14]. It is therefore relevant to investigate them in a neutral spine posture during isometric contractions.

Surface electromyography (EMG) is a technique frequently used to examine forces and activation patterns during direct and indirect perturbations to the head [12–14]. EMG allows indirect estimation of internal loads via Newton’s laws of motion when the EMG-force relationship is characterized, which is essential to injury mechanism elucidation, as direct measurements of internal loads are both infeasible and ethically undesirable. It is generally accepted that the EMG-force relationship is positive; however, reported relationship shapes vary and need to be established separately for individual muscles due to the many factors that influence EMG measurements [16, 17]. To our knowledge, five studies have, to various extents, examined the EMG-force relationship of the neck during isometric contractions in a neutral spine posture [18–22]. Reported results have incongruities, but suggest a rectilinear relationship for measurements over sternocleidomastoids and semispinalis capitis, and a curvilinear relationship over splenius capitis. However, because none of these studies provided models, information from the EMG-force relationship cannot be extrapolated. In addition, methodological limitations such as small sample sizes, insufficient statistical analyses, contraction intensities below 50% of maximal force, or ramp contractions, which incorporate incremental speed of contraction intensity as a confounding variable, impede the results’ dependability. Hence, further investigation of the neck muscle EMG-force relationship is necessary to facilitate interpretation of studies based on EMG measurements and to provide a basis for accurate biomechanical models. To adjust for imprecision in force estimation, it is also essential to assess the EMG-force relationship’s reliability. This study therefore examined the neck muscle EMG-force relationship and quantified this relationship’s test-retest reliability.

## Methods

### Research Design and Subjects

In three trials distributed over two days within a two-week period, neck muscle EMG-force relationships were examined across seven submaximal contraction intensities during sustained isometric constant-force contractions for neck flexion and neck extension. Following approval by the Regional Committee for Medical Research Ethics, a convenience sample of eighteen participants was recruited through personal communication. Inclusion criteria were being healthy and aged 18–65 years; exclusion criteria were neck or shoulder pain within the last three months and known patch allergy. Nine males and nine females with a mean (SD; min-max) age of 29 (7; 20–48) years, height of 1.70 (0.08; 1.53–1.81) m, and weight of 66 (10; 45–84) kg enrolled for participation and signed a written informed consent. Eleven participants reported that they had trained for muscle strength or cardiovascular fitness more than three times per week over the past 12 months. All participants were requested to refrain from alcohol and physical training of the neck and shoulders for 72 h preceding measurements.

### Protocol

**Fig. 1:**
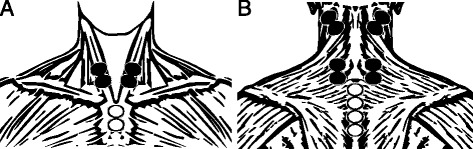
Electrode placement for the anterior (**a**) and the posterior (**b**) neck. *Filled* and *unfilled circles* mark detection and reference electrodes, respectively

EMG activity was monitored bilaterally in three areas (Fig. 1): the anterior neck (AN) – centered on the belly of the sternocleidomastoid muscle, approximately one third of the length above the sternal attachment [21], the upper posterior neck (UPN) – centered over the most superficial splenius capitis muscle area, between the sternocleidomastoid and upper trapezius muscles [19–21], and the lower posterior neck (LPN) – over the lower semispinalis capitis, 20 mm lateral to the median line in level with the C7 spinous process [20]. Reference electrodes were placed over sternum and spinous processes. In accordance with current recommendations [23], electrode placement areas were meticulously shaved, abraded, and disinfected with alcohol. Disposable, self-adhesive, pre-gelled Ag/AgCl electrodes with a conductive diameter of 10 mm (Blue Sensor N-00-S, Medicotest A/S, Ølstykke, Denmark) were attached pairwise with an inter-electrode distance of 20 mm, and secured with surgical tape. Skin potential offset was allowed to stabilize for 20 min preceding measurements, after which electrode-skin impedance was consistently below 5 kΩ. To obtain temporally synchronized data, differentially amplified EMG (bandwidth 8–500 Hz, input impedance = 10 GΩ, CMRR = 110 dB at 50 Hz, input noise <1.6 μV_RMS_, gain = 305) and force signals (TB5 tension load cell, Lahti Precision Ltd, Lahti, Finland; maximal load = 500 kg, error <0.02%, sensitivity <0.1%) were digitized by a 14-bit A/D converter with a sampling frequency of 1000 Hz and stored in an integrated system (Biomonitor ME6000, Mega Electronics Ltd, Kuopio, Finland).

**Fig. 2:**
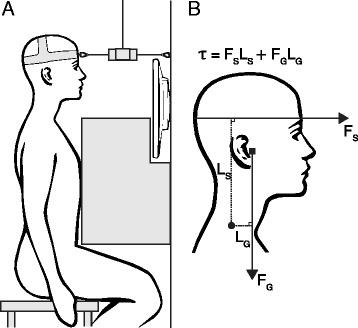
Schematic diagrams of the data sampling setup and torque calculation. **a** shows body positioning during neck extensions with a square block stabilizing the spine in a neutral posture, arms hanging slack beside the torso, and legs placed to prevent them from aiding during trials. A wall-attached force transducer, supported by a sling from the roof, is fixed to a strap that is level with the supraorbital ridge. Visual feedback is provided by a monitor on the wall. During neck flexions, subjects are reversed 180°, facing an additional monitor (not shown in figure). **b** shows the neck extension torque (*τ*) formula along with a visual description of its variables: the resistance force of the strap (*F*_S_), the force on the center of mass of the head and neck (*filled square*) due to gravitational acceleration (*F*_G_), and their respective lever arms (*l*_S_ and *l*_G_) originating from the axis of rotation between the C7–T1 spinous process (*filled circle*)

Subjects were seated in a standardized position on a fixed, height-adjustable stool, with a rigid square block stabilizing the torso in a neutral spine posture, and the extremities placed to prevent them from aiding during trials (Fig. 2a). A rigid strap connected to a fixed force transducer was placed over the supraorbital ridge and reference values were acquired through three maximal voluntary contractions for each contraction direction, during which subjects pushed their head against the strap. Subjects were carefully instructed as to the correct technique. To obtain accurate reference values, maximal contractions were rehearsed before trials, verbal encouragement and visual feedback of the force output was provided during trials, and the highest 1-s average of the highest contraction was selected. Reference values were used to calculate submaximal target contraction intensities equivalent to 5, 10, 20, 30, 50, 70, and 90% of maximal force.

Following a 30-min rest, participants met target contraction intensities aided by live visual feedback of produced force. When subjects did not reach target intensities, they were permitted an additional trial. Software malfunction resulted in two cases failing to meet this criterion: one being 18% instead of 20%, and one 26% instead of 30%; both included in the analyses below. Total time for the submaximal protocol was 40 min, followed by a one-hour break with electrodes retained, after which the submaximal contraction procedure was repeated. The same 40-min sampling procedure was repeated again 5–14 days after the first day, with identical preparatory steps. To avoid systematic bias due to muscular fatigue, contraction direction and intensities were conducted in random order, sustained for approximately five seconds, and separated by 2-min rest periods. Data sampling took place in a university motion laboratory, and was carried out by a trained physiotherapist with prior experience of the protocol.

### Data Management and Statistics

Data management was conducted in MATLAB (v8.1, The MathWorks Inc., Natick, MA, USA). Unprocessed signals were visually inspected in time and frequency domains. EMG and force signals were then respectively band-passed at 20–400 Hz and low-passed at 25 Hz, with a fourth-order zero phase Butterworth filter. Next, EMG signals were full-wave rectified, and both EMG and force signals were smoothed by a centered 1-s moving average with a 1-ms lag. The signal-to-noise ratio was calculated as the ratio of desired EMG signal power to the noise signal power obtained during rest to provide a measure of EMG signal quality. Automated functions were employed to extract submaximal values from the smoothed data by selecting the most stable second of force data within ±5% of the target intensity, followed by extraction of the coinciding EMG amplitude. All selected values were visually controlled to confirm their adequacy. Torques were calculated for neck flexion and extension from force data, and for gravity on the mass of the head and neck [20], using a constant of 7.9% of body weight [3]. Gravitational torque was then subtracted from neck flexion and added to neck extension. The torque calculation details are provided in Fig. 2b. Because target contraction intensities did not account for gravity, data below 20% of maximal target force in some cases (*n* = 16 for 5%, *n* = 6 for 10%, and *n* = 1 for 20% of target intensity) corresponded to neck flexion torques below zero. These values were discarded in the following analyses, since it was uncertain whether they were related to the examined exertion or to gravity alone. Submaximal force and EMG values were normalized as the percentage of maximal force (% MVC), and maximal voluntary excitation (% MVE), respectively. Finally, to decrease random bias related to measurement error [24], EMG data averaged between both muscle sides and trials were used for EMG-force relationship analyses, whereas EMG data averaged between muscle sides were used for the reliability analysis.

Statistical analyses were conducted in R (v3.1.3, R Core Team 2015, R Foundation for Statistical Computing, Vienna, Austria). EMG-force relationships were visually inspected and modeled with general linear mixed-effects regression via a top-down strategy [25]: following selection of fixed effects, random effects were iterated one by one, and last, fixed effects were reduced until the most parsimonious model was identified. Maximum likelihood estimation was used to fit models in which the number of fixed effects differed, while restricted maximum likelihood estimation was used to fit models in which random effects differed. Akaike’s information criterion and Schwarz’s Bayesian information criterion aided in the model selection [26], and the Pratt-adjusted coefficient of determination [27, 28] and the root mean square error [29] were used as measures of the models’ fit to the data. Statistical assumptions were assessed visually [25]. The influence of antagonistic muscle activity was tested for all sampling areas.

Test-retest reliability of individual subjects’ modeled EMG-force relationships were examined within and between days across sampling areas. The final EMG-force relationship models were refitted for each of the three trials, and the participant-specific regression lines were extracted from the refitted models and compared between trials per percentile across the contraction intensity range. Changes in the mean were presented graphically. An intraclass correlation coefficient (ICC) based on a single measurement two-way random effects model of absolute agreement was utilized to examine test-retest correlation [30, 31], and the standard error of measurement (SEM; i.e., the within-subject standard deviation between trials) [31] was used to investigate within-subject variation. Alpha was set at 0.05 for all analyses.

## Results

### Non-Normalized Force and EMG Data

Noticeable intersex differences were observed for maximal force with average female/male force ratios of 0.5 for neck flexion and 0.6 for neck extension. In absolute numbers, mean (SD) measured torques of 16 (5) Nm vs. 34 (13) Nm for flexion and 34 (4) Nm vs. 60 (14) Nm for extension were registered for females and males, respectively.

Signal-to-noise ratio calculations revealed that LPN had a markedly lower signal amplitude than other sampling areas. The mean (SD; min-max) reference contractions’ signal-to-noise ratios were 48 (6; 38–61) dB for AN, 44 (5; 32–54) dB for UPN, and 31 (3; 22–36) dB for LPN.

### EMG-Force Relationship

Visual inspection of individual subjects’ EMG-force curves suggested multiple patterns within the same sampling area, with observations noticeably more homogenous for UPN and LPN than for AN. Two-thirds of all cases appeared rectilinear for AN. In contrast, for UPN and LPN, approximately one third of the curves had a distinct positively oriented quadratic shape, one third suggested a piecewise linear pattern with a breakpoint at roughly 25% MVC, and one third could fit both profiles.

**Fig. 3:**
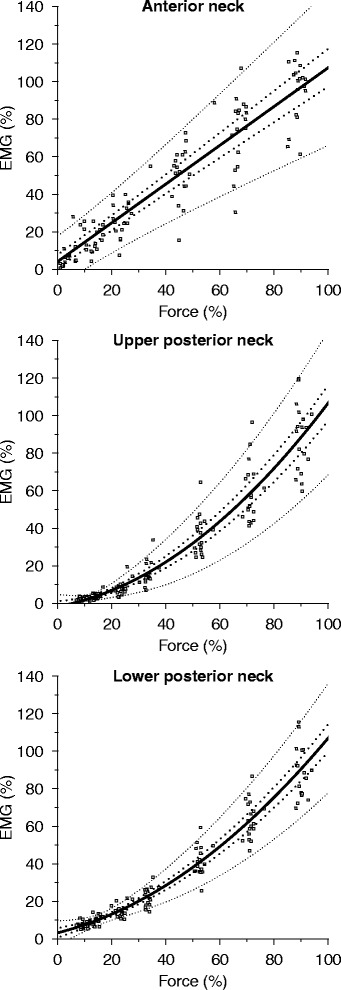
Final EMG-force relationship models. *Solid lines* denote the fitted regression line on the sampled data points (*gray squares*). Inner and outer dotted lines mark the 95% confidence interval of the fitted lines computed via the delta method, and the 95% prediction interval generated via parametric bootstrap with 10,000 replicates, respectively

Figure 3 displays the fitted models juxtaposed on the sampled data, while Table 1 provides details for the final models. First-order models had the best fit for AN, whereas second-order models had the best fit for UPN and LPN. Goodness-of-fit indices were consistent, in that incorporating the intercept and slope as random effects (i.e., allowing by-subject variation of the intercept and slope) substantially improved models across all sampling areas (Table 1); no other random effects improved final models. Point estimates of the adjusted conditional coefficient of determination and root mean square error both demonstrated excellent model fit for all sampling areas, with 97–98% of the variance accounted for by the models, and absolute errors of 4–5% MVE. Mean antagonistic EMG activity during neck flexion was 20–42% and 17–133% for UPN and LPN, respectively, relative to AN EMG activity, whereas that of AN during neck extension was 4–30% and 5–10% relative to UPN and LPN activity, respectively. With the exception of the UPN activity during neck flexion, which had a U-shaped pattern, relative antagonistic activity decreased with contraction intensity. Antagonistic muscle activity did not remain in any of the final models, as no significant effect was observed with its inclusion.

**Table 1:** Final EMG-force relationship models

	Anterior neck		Upper posterior neck		Lower posterior neck	
	Est	95% CI	Est	95% CI	Est	95% CI
FE						
*b*_*0*_	4.2 × 10^0^	0.6 × 10^0^, 7.8 × 10^0^	−1.7 × 10^0^	−4.3 × 10^0^, 1.1 × 10^0^	3.3 × 10^0^	0.9 × 10^0^, 5.7 × 10^0^
*b*_*1*_	1.0 × 10^0^	0.9 × 10^0^, 1.1 × 10^0^	2.7 × 10^−1^	0.1 × 10^−1^, 4.2 × 10^−1^	3.6 × 10^−1^	2.3 × 10^−1^, 4.9 × 10^−1^
*b*_*1*_^*2*^	–	–	8.2 × 10^−3^	6.9 × 10^−3^, 9.5 × 10^−3^	6.8 × 10^−3^	5.7 × 10^−3^, 7.8 × 10^−3^
RE						
σ_*b0*_	7.4 × 10^0^	4.6 × 10^0^, 10.4 × 10^0^	2.8 × 10^0^	0.8e^0^, 4.5e^0^	3.3 × 10^0^	1.6 × 10^0^, 5.1 × 10^0^
σ_*b1*_	2.0 × 10^−1^	1.3 × 10^−1^, 2.8 × 10^−1^	2.3 × 10^−1^	1.4 × 10^−1^, 3.1 × 10^−1^	1.8 × 10^−1^	1.2 × 10^−1^, 2.4 × 10^−1^
σ_ε_	4.3 × 10^0^	3.6 × 10^0^, 5.1 × 10^0^	4.6 × 10^0^	4.1 × 10^0^, 5.4 × 10^0^	3.7 × 10^0^	3.2 × 10^0^, 4.3 × 10^0^
R^2^M_adj_	0.84	0.80, 0.85	0.89	0.87, 0.90	0.92	0.91, 0.93
R^2^C_adj_	0.98	0.97, 0.98	0.97	0.95, 0.97	0.98	0.97, 0.98

b_0_, intercept. b_1_, first-order term (slope). b_1_^2^, second-order term (curvature). σ_b0_, standard deviation of the intercept. σ_b1_, standard deviation of the slope. σ_ε_, standard deviation of the within-subject residual. R^2^M_adj_, adjusted marginal coefficient of determination. R^2^C_adj_, adjusted conditional coefficient of determination

*Est* point estimate, *95% CI* 95% confidence interval, *FE* fixed effects, *RE* random effects

### EMG-Force Relationship Reliability

**Fig. 4:**
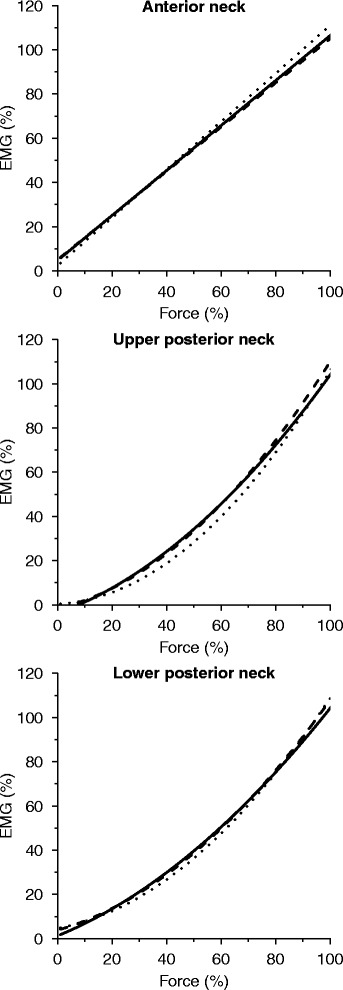
By-trial fitted regression lines. *Solid*, *dashed*, and *dotted lines* denote by-trial fitted regression lines for the first day’s morning session, the first day’s afternoon session, and the second day’s morning session, respectively

**Fig. 5:**
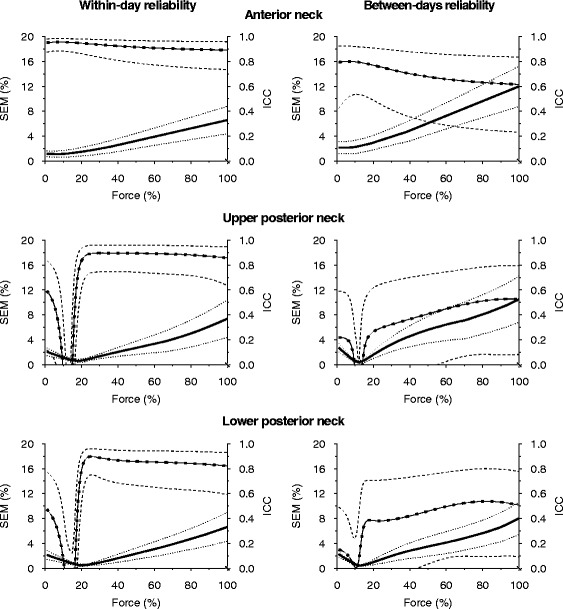
Test-retest reliability of the EMG-force relationship over the full force range. *Solid lines* denote point estimates of the standard error of measurement (SEM), and *dotted lines* its 95% confidence interval (CI). *Lines with squares marks* point estimates of the intraclass correlation coefficient (ICC), and *dashed lines* its 95% CI

To provide a measure of the between-trial changes in the mean, Fig. 4 shows the fitted line for each of the three trials. In absolute numbers, mean force differences were below 6% for all sampling areas, with UPN having the largest between-trial discrepancies (within-day: AN ≤ 1%, UPN ≤ 3%, LPN ≤ 4%; between-day: AN ≤ 3%, UPN ≤ 6%, LPN ≤ 6%). Figure 5 shows test-retest reliability estimates of individual participants’ fitted EMG-force relationship regression lines within and between days through 0–100% of MVC. Both ICC and SEM estimates tended to be unstable from 0–20% MVC. From 20–100% MVC, mean ICC for within-day was 0.79 or higher (AN ≥ 0.89, UPN ≥ 0.83, LPN ≥ 0.79), whereas it for between-day was 0.27–0.61 (AN ≥ 0.61, UPN ≥ 0.27, LPN ≥ 0.38), with the lower 95% confidence boundary frequently below zero. Over the same force range, the mean SEM was below 12% of the corresponding EMG magnitude for all sampling areas (within-day: AN ≤ 6%, UPN ≤ 9%, LPN ≤ 6%; between days: AN ≤ 12%, LPN ≤ 10%), with the exception of the UPN between days, for which this applied above 42% MVC.

## Discussion

This study examined neck muscles’ EMG-force relationships and assessed the modeled relationships’ test-retest reliability. The main findings were that relationships were rectilinear for the anterior neck (AN) and the upper shoulders (US), and curvilinear for the upper (UPN) and lower posterior neck (LPN). Group-level relationships were similar between trials, and relationship reliability for individual participants was acceptable over most contraction intensities, albeit less reliable between than within days.

Consistent with previous findings [18, 21], AN displayed a near one-to-one rectilinear relationship between EMG and force, and the model provided a good data fit which accounted for 98% of the total variance. This model can therefore aid interpretation of EMG studies when inferences are to be linked to force on a group level and have an accuracy within a 4% mean error margin. In contrast, both UPN and LPN displayed curvilinear relationships and force values were considerably higher than EMG values over most contraction intensities. Assuming a one-to-one relationship for these muscles would therefore lead to underestimated force values if inferences were to be drawn from EMG data. The final models for UPN and LPN had an excellent fit to the data, with 97–98% of the total variance accounted for, and an accuracy level within an average 4–5% error margin. Our UPN results are in agreement with the findings of three prior investigations [19, 20, 22], whereas a fourth study reported a rectilinear relationship [21]. The standardized weights used to derive the latter study’s results, however, corresponded to a limited range of low intensities in our study, for which, in isolation, the relationship could also be interpreted as rectilinear. In sum, our results for UPN are supported by findings in the literature and therefore likely reflect the actual EMG-force relationship for the electrode placement area. For LPN, one previous study reported a curvilinear relationship [20], whereas three prior studies reported rectilinear relationships [18, 19, 21], contrariwise to our results. This incongruity is probably a result of the limited contraction range investigated in two of these studies [19, 21]. Indeed, upon visual inspection of the LPN EMG-force relationship curves in the 0–50% range (Fig. 3), it is apparent that a linear curve could be fitted to the data and likely provide an accurate predictive measure, but the measures could not be extrapolated beyond that range, whereas our curve provides predictive values over nearly the whole force range. In the third study [18], the few subjects (five) combined with the limited number of investigated contraction intensities (three) is unlikely to have provided the proper resolution to elucidate the curvilinearity of the relationship. In the study in agreement with our results, contractions were measured over 3–75% range for 10 females. The larger contraction range examined likely provides the explanation for the agreement. In sum, our results agree with those presented in the literature, with the difference that our measurements over nearly the full contraction range combined with our sample size of 18 allowed us to elucidate the EMG-force relationship over a larger interval and to a more specific degree. Visual inspection of LPN data suggested that piecewise linear models may have a better fit for some individuals. However, a greatly increased complexity rendered the implementation of such models impractical.

Overall EMG-force relationships displayed a maximum 6% between-trial discrepancy, with the rectilinear model being more similar between trials than curvilinear models across most of the contraction range (Fig. 4). No clear systematic biases accounting for these discrepancies were observed, and we therefore assume that they are the result of random variation which needs to be considered when inferences are to be drawn from EMG to force on group-level test-retest investigations of similar sample sizes. No analogous data exists for EMG-force relationship reliability on an individual level, and guidelines for relevant point estimate evaluation have therefore not yet been established. However, universal guidelines have previously defined ICC estimates of 0.00–0.39, 0.40–0.59, 0.60–0.74, and 0.75–1.00 as poor, fair, good, and excellent, respectively [32], and SEM estimates for acceptable reliability range from 7.5–20% of the measured variable, with 15% being the most common cutoff [33]. Interpreting ICC and SEM point estimates in light of these guidelines, within-day and between-day reliability for individual AN EMG-force relationships was excellent over 9 and 12% MVC, respectively. For UPN and LPN, within-day reliability was excellent over 20% MVC, whereas between-day reliability was fair over 58 and 33% MVC, respectively. Hence, reliability was unacceptably low at low contraction intensities, and consistently higher within rather than between days, which supports that individual EMG-force relationships are stable over most contraction intensities within the same day, but can vary considerably for between-day measurements. Universal standards should be interpreted with caution, as they may have considerable limitations when generalized. ICC estimates are context-specific, as magnitude of ICC is inversely related to sample homogeneity [30, 31]. In this study, the mean between-subject coefficient of variation following 20% MVC ranged from 17–27% across sampling areas and was 10–18% lower from first- to second-day trials. The lower second-day sample variance therefore provides a partial explanation for the large reduction in ICC estimates between days that was not consistently reflected in the SEM estimates. In contrast to ICC, which suggested that relationship agreement was unacceptably low between trials over certain force intensities, SEM showed that absolute differences remained within an acceptable level. A less conservative interpretation based on absolute between-trial differences alone therefore suggests acceptable reliability for UPN between days over 58% MVC and for LPN past 10% MVC both within and between days. AN and LPN can therefore likely be reliably measured both within and between days for all but the lowest contraction intensities, whereas UPN is limited to within-day measurements over 20% MVC and between-day measurements over 28% MVC.

This study has some limitations. The measurements obtained should be construed as location-specific rather than muscle-specific due to the various factors influencing EMG measurements [17]. One major factor was EMG activity contributions due to co-contraction of adjacent muscles [17], which was of particular concern for the UPN measurements, owing to its proximity to the antagonist AN. In contrast, force measurements were limited in that only the net force effect was obtained, while antagonistic activity added to internal loads; however, including antagonistic activity as a covariate in regression models did not improve them, thus indicating their influence to be inconsistent. The complexity of the neck muscle anatomy [34] rendered precise electrode placement challenging, and likely decreased signal quality in some measurements. LPN signal amplitude was lower relative to other sampling areas and is attributable to the tendinous structures below the electrodes. The high signal stability and good regression model fit, however, support that valuable information can be extracted from this location, and it is questionable whether upwards relocation of electrodes would improve signal quality, as prior research has also reported low signal amplitudes on posterior muscles at the C4-level [35].

The EMG-force relationship models provided permit inferences to be drawn from isometric neck muscle EMG measurements to force on a group level, and enable cervical loads to be estimated during activities in EMG-driven biomechanical models. In total, three force estimation models were derived: one for neck flexion and two for neck extension. Of the two latter models, the higher accuracy of the LPN model makes it the better choice for estimating neck extension forces. In addition, the between-trial differences in mean EMG-force relationships suggest that the models are stable over time. They do not, however, allow prediction of individual values, with maximum prediction errors ranging between approximately 30–60% force across sampling areas. Further, individual-level relationships showed insufficient reliability over the lower parts of the contraction range, which prevents meaningful analyses from being made for individuals over those intensities. Figure 3 allows force to be estimated visually, while the regression coefficients provided in Table 1 enable precise force calculation. In contrast to many laboratory studies, factors such as temperature, sound level, time of measurement, surrounding crowd, and exact electrode placement were allowed to deviate between trials to render results more representative of field measurements. Combined with the recruitment criteria, the sample’s demography, and neck strength being similar to normative values [4], our results may be generalized to relatively fit and healthy adults between 20–50 years.

## Conclusions

Of the studied models, a rectilinear model was best suited for the anterior neck while curvilinear models were best suited for the upper and lower posterior neck. All models had small between-trial variation in the mean and we therefore conclude they can be reliably used to estimate force from EMG on a group level. Models did not have sufficient accuracy for force prediction from single EMG values. However, EMG-force relationships for individual participants typically had acceptable reliability over most of the contraction range both within and between days, suggesting that individual-level relationships remain stable over time. Provided models enable inferences to be drawn from EMG to force on a group level, and reliability estimates facilitate adjustment for measurement imprecision.

## Abbreviations

AN: Anterior neck
EMG: Electromyography
LPN: Lower posterior neck
UPN: Upper posterior neck
